# Glioblastoma Multiforme: Sensitivity to Antimicrobial Peptides LL-37 and PG-1, and Their Combination with Chemotherapy for Predicting the Overall Survival of Patients

**DOI:** 10.3390/pharmaceutics16091234

**Published:** 2024-09-22

**Authors:** Alexander N. Chernov, Sofia S. Skliar, Alexander V. Kim, Anna Tsapieva, Sarng S. Pyurveev, Tatiana A. Filatenkova, Marina V. Matsko, Sergey D. Ivanov, Olga V. Shamova, Alexander N. Suvorov

**Affiliations:** 1 World-Class Research Center “Center for Personalized Medicine”, Federal State Budgetary Institution of Science “Institute of Experimental Medicine”, 197376 Saint Petersburg, Russia; tsapieva.an@iemspb.ru (A.T.); lero269@gmail.com (T.A.F.); oshamova@yandex.ru (O.V.S.); alexander_suvorov1@hotmail.com (A.N.S.); 2 Federal State Budgetary Educational Institution of Higher Education, St. Petersburg State Pediatric Medical University of the Ministry of Health of Russia, 194100 Saint Petersburg, Russia; spb@gpma.ru; 3 Children’s Neurosurgical Department No. 7, Almazov Medical Research Centre, 197341 Saint Petersburg, Russia; sklyar_ss@almazovcentre.ru; 4 Laboratory of Neurooncology of Polenov Neurosurgical Institute, Almazov National Medical Research Centre, 197341 Saint Petersburg, Russia; kim_av@almazovcentre.ru; 5 Scientific Department of State Budgetary Healthcare Institution Saint-Petersburg Clinical Scientific and Practical Center for Specialized Types of Medical Care (Oncological) named N.P. Napalkov, 197758 Saint Petersburg, Russia; marinamatsko@mail.ru; 6 Department of Oncology, Medical and Social Institute, Saint-Petersburg University, 199034 Saint Petersburg, Russia; 7 FGBU N.N. Petrov National Medical Research Center of Oncology, Ministry of Health of the Russian Federation, 197758 Saint Petersburg, Russia; sdivanov44@mail.ru; 8 Saint Petersburg State University, 199034 Saint Petersburg, Russia

**Keywords:** glioblastoma, LL-37, PG-1, cytotoxicity, chemotherapy drugs, combinations of LL-37, PG-1 with chemotherapy, overall survival of GBM patients

## Abstract

**Background/Objectives:** Glioblastomas (GBMs) are the most malignant and intractable of all cancers, with an unfavorable clinical prognosis for affected patients. The objective was to analyze the sensitivity of GBM cells to the antimicrobial peptides (AMPs) cathelicidin (LL-37) and protegrin-1 (PG-1), both alone and in combination with chemotherapy, to predict overall survival (OS) in the patients. **Methods:** The study was conducted on 27 GBM patients treated in the neurosurgical department of the Almazov Medical Research Centre (Saint Petersburg, Russia) from 2021 to 2024. The cytotoxic effects of chemotherapy, AMPs, and their combinations on brain tumor cells were assessed by an MTT assay using a 50% inhibitory concentration (IC_50_). **Results:** In GBM cells from the patients, LL-37 and PG-1 exhibited strong anticancer effects, surpassing those of chemotherapy drugs. These LL-37 and PG-1 anticancer effects were associated with a statistically significant increase in life expectancy and OS in GBM patients. These findings were confirmed by experiments on rats with C6 glioma, where the intranasal administration of LL-37 (300 μM) and PG-1 (600 μM) increased the life expectancy of the animals to 69 and 55 days, respectively, compared to 24 days in the control group (HR = 4.139, *p* = 0.0005; HR = 2.542, *p* = 0.0759). **Conclusions:** Additionally, the combination of LL-37 and PG-1 with chemotherapy drugs showed that a high IC_50_ of LL-37 with cisplatin (cutoff > 800 μM) in GBM cells was associated with increased life expectancy (19 vs. 5 months, HR = 4.708, *p* = 0.0101) and OS in GBM patients. These combinations could be used in future GBM treatments.

## 1. Introduction

Oncological diseases currently present significant social and economic challenges [1]. According to estimates from the World Health Organization (WHO) and the International Agency for Research on Cancer (Globocan), in 2020, there were 19,292,789 new cases of cancer and 9,958,133 cancer-related deaths worldwide [2]. The prevalence of primary brain and central nervous system tumors was assessed at 308,102 cases in 2020 [3]. Glioblastoma multiforme (GBM) is the most frequent and aggressive brain tumor in adults, accounting for 85–90% of all human brain tumors [4]. The clinical prognosis for patients with these tumors is unfavorable, with a life expectancy of only 14.6 months and a 100% relapse rate [5].

Current GBM treatment includes neurosurgery, radiotherapy, and chemotherapy with temozolomide (TMZ) [4,6]. The most effective chemotherapy drugs for GBM treatment are TMZ, cisplatin (CSP), carboplatin (CARB), etoposide (ETO), doxorubicin (DOX), and pembrolizumab [6,7,8,9,10]. Several factors contribute to the failure of GBM therapy, including the heterogeneity of GBMs, encompassing GBM stem cells (GSCs) that are often resistant to radiochemotherapy [11]. This heterogeneity is also due to the expression of various surface and intracellular markers that contribute to the development of multidrug resistance, tumor metastases, and relapses in treated patients [12].

A new approach to cancer therapy may involve the use of cationic antimicrobial peptides (AMPs) or their combinations with chemotherapy drugs. To date, more than 23,253 AMPs have been identified [13]. Most AMPs are composed of 12–50 amino acid molecules with a high content of arginine and/or lysine. AMPs exhibit different structures and possess antimicrobial, antiviral, and immunomodulatory activities, with some demonstrating anticancer effects [14,15]. Anti-cancer AMPs have already been identified [15]. Their effects depend on the structural characteristics and mechanisms of each peptide, as well as on the characteristics of mammalian cells and microorganisms. For example, microorganisms have more negative membrane potential than mammalian cells [16].

In this study, we focused on two neutrophilic peptides with different structures: human cathelicidin (LL-37) with an α-helical structure, and pig protegrin-1 (PG-1) with a β-hairpin conformation [17]. Cathelicidin LL-37 shows multidirectional tissue-specific actions: increased expression of the cathelicidin antimicrobial peptide (*CAMP*) gene and its secretion are associated with the progression of lung epithelial adenocarcinoma (A549), breast cancer (MCF-7, MDA-MB-435s, MDA-MB-231), and pancreas, colon, hepatoma, and oral squamous cell carcinoma cells [18,19,20,21,22,23]. Conversely, the expression of the *CAMP* gene and LL-37 secretion levels are significantly inhibited in stomach cancer, HT-29 and HCT116 colon cancers, chronic lymphocytic leukemia, U937 lymphoma, and SH-SY5Y neuroblastoma cells [24,25,26,27,28]. 

Recently, we also demonstrated that LL-37 induces cell death in C6 and U251 glioma cells [29,30,31]. However, at high concentrations, these peptides are toxic to human cells, which poses a challenge regarding their use in medical practice [31]. One potential solution to this challenge is reducing the effective concentrations of AMPs. This reduction can be achieved through the synergistic anticancer effects of combining LL-37 and PG-1 with chemotherapy drugs. The sensitivity of GBM to LL-37 and PG-1 antimicrobial peptides, as well as their combinations with chemotherapy, for predicting patient survival remains unexplored. 

The aim of the study was to analyze the sensitivity of GBM cells to the antimicrobial peptides LL-37 and PG-1, both alone and in combination with chemotherapy, to predict the OS of GBM patients.

## 2. Materials and Methods

### 2.1. Clinical Characteristics of Patients

The study involved 27 GBM patients treated in the neurosurgical department of the Almazov Medical Research Centre (Saint Petersburg, Russia) from 2021 to 2024. All patients provided informed consent and underwent surgical resections, MRIs in the neurology department, and histological verification in the pathomorphological department. The investigation was approved by the local Ethics Committee of the Institute of Experimental Medicine (No. 6/20, from 21 October 2020). 

### 2.2. Cell Culture

Biopsies of tumors were collected from the patients. Primary GBM samples were cut into small (2–4 mm) pieces under sterile conditions and washed to remove blood and necrotic elements. Tumor cells were dissociated in a 0.25% trypsin solution in EDTA for 5–10 min at 37 °C and cultured at a concentration of 1 × 10^4^ cells per well in 96-well flat-bottomed plates (TPP, Trasadingen, Switzerland). The GBM cells were cultivated under normal conditions: Dulbecco’s modified Eagle medium (DMEM) with 10% fetal bovine serum (Sigma-Aldrich, St. Louis, MO, USA) and gentamicin sulfate 10^−4^ g/mL (Shandong Weifang Pharmaceutical Factory Co., Weifang, China) at 37 °C and 5% CO_2_ for 1–2 days [32,33].

### 2.3. MTT Assay

The anticancer effects of the chemotherapeutic drugs, LL-37, PG-1, and their combinations, on GBM cells (1 × 10^4^ cells/well) were studied using the MTT test in 96-well plates [34]. We diluted 2-fold of LL-37 and PG-1 and 2–10-fold of chemotherapy drugs in 50 μL of DMEM into each well of the plate. To investigate the combinations of the two drugs, 25 μL of each substance was introduced at the appropriate dose. All doses of the drugs and combinations were tested three times. For a positive control, DMEM was added to the wells with GBM cells instead of the drug. As a negative control, DMEM was introduced to the empty wells of the plate. Plates with GBM cells were maintained for 24 h at 37 °C with 5% CO_2_. The next day, MTT solution (25 μL, 5 mg/mL) was put into the cell culture wells and incubated for 3 h under the same conditions. After incubation, 50 μL of isopropanol alcohol with 0.04 N HCl was introduced to all wells and mixed. The solution’s optical density was measured at 540 nm as a test and with 590 nm wavelengths as background, using a SpectraMax 250 plate spectrophotometer and SoftMax Pro 5.2 software (Molecular Devices, San Jose, CA, USA). The cytotoxic capacity of the drugs was calculated as the percentage of dead cells, based on comparing the optical density of GBM cell wells under positive (100% viable cells) and negative (0% viable cells) controls using Formula (1):

(1) $$\mathrm{DC}(\%)=\frac{\mathrm{OD}(\mathrm{control})-\mathrm{OD}(\mathrm{test})}{\left(\mathrm{OD}(\mathrm{control}\right)-\mathrm{OD}(0\%\;\mathrm{VC}))}\times 100$$

where DC (%) is the percentage of dead cells in the test wells; OD (test) is the optical density of the test drug at a given dose; and OD (0% VC) is the average optical density of empty wells with a culture medium.

### 2.4. Determination of IC_50_ Dose, Combination Index and Combination Effects

To study the anticancer effects of LL-37, PG-1, chemotherapy drugs, and their combinations on GBM cells, the dose of 50% inhibition of cell viability (IC_50_) was calculated. Tumor cells were incubated with LL-37, PG-1, CIS, CARB, DOX, TMZ, and ETO at various concentrations, Table 1. 

AMPs, chemotherapeutic drugs, and their combinations were analyzed using nonlinear regression with Origin Pro 8.5 software. The IC_50_ of each component of the combination was determined, based on fixed proportions for each pair of substances, depending on the IC_50_ values of the combination, using Formula (2):

IC_50_ (each component of the combination) = IC_50_ (combination) × W (2)

where W is the proportion of each chemotherapy drug in the combination.

### 2.5. Wistar Rat Intracerebral C6 Glioma Model

The experiments were performed on 15 Wistar male rats (with a body weight of 200–300 g), which were housed in the vivarium of the Institute of Experimental Medicine (Saint Petersburg, Russia). All rats were intraperitoneally anesthetized with chloral hydrate (10%, 4 mL/kg) and fixed in a prone position on a rat stereotaxis. For all rats, we performed neurosurgery with the introduction of 10^6^ C6 glioma cells into 10 µL of saline. The rats were separated into two groups: control (*n* = 5) and tentative (*n* = 10). Control rats were intranasally injected with 20 µL of saline twice a week. In the testing groups, 20 µL of LL-37 (*n* = 5, 300 µM) and PG-1 (*n* = 5, 600 µM) were intranasally administered twice a week. The weight, tumor size, and survival of the rats were determined. The study was approved by the local ethics committee at the Institute of Experimental Medicine (No. 6/20 dated 21 October 2020) [35].

### 2.6. Reagents

The following reagents were used in the study: human cathelicidin LL-37 (Cat. No. AS-61302, 1 mg, Anaspec, Fremont, CA, USA); protegrin-1 (Cat. No. AS-64819-05, 0.5 mg, Anaspec, Fremont, CA, USA), gentamicin sulfate (solution of 40 mg/mL, Shandong Weifang Pharmaceutical Factory Co., Liaocheng, China); Doxorubicin-LANS^®^ (solution of 2 mg/mL, Veropharm, Moscow, Russia); Carboplatin-LANS^®^ (solution of 10 mg/mL, Veropharm, Moscow, Russia); temozolomide (Temodal capsules, 100 mg, Orion Pharma, Espoo, Finland); Cisplatin-LANS^®^ (solution of 0.5 mg/mL, Veropharm, Moscow, Russia); etoposide (solution of 20 mg/mL, Ebewe Pharma, Unterach am Attersee, Austria); and a streptavidin–biotin–peroxidase kit (LSAB2, Dako, Glostrup, Denmark). 

### 2.7. Statistical Analysis

All experiments were performed at least in triplicate. The statistical significance of differences between the means of different treatments and their respective control groups was determined using Student’s *t*-test. Multiple differences between several groups were calculated using a one-way ANOVA test. Data were presented with the standard deviation and were considered significant at *p* < 0.05. To compare the differences between two independent groups with a small number of samples (*n* < 30), the nonparametric Mann–Whitney U-test was used [36]. Descriptive statistics, an ANOVA test, and OS analysis were performed using GraphPad Prism software (version 8.01, 09.21.2020, San Diego, CA, USA).

## 3. Results

### 3.1. Personalized In Vitro Cytotoxic Effects of Chemotherapy Drugs, LL-37, PG-1, and Their Combinations on Patients’ GBM Cells Using the MTT Assay

Initially, we evaluated the anticancer effects of LL-37 and PG-1, comparing them to the effects of the chemotherapy drugs on GBM cells, as shown in Table 2.

Table 2 shows that GBM cells from the patients exhibited multidrug resistance to chemotherapy drugs. Specifically, GBM cells from patients 11961, 6770, 18871, 114495, 18871, 39114, 40906, 48307, 9439, and 7593 were resistant to all five tested drugs. GBM samples from patients 11081 and 62642 showed resistance to four drugs: CARB, TMZ, CIS, and ETO. GBM cells from patients 55068, 25873, and 49142 displayed resistance to DOX, CARB, TMZ, and CIS. GBM samples from patients 1401 and 48307 showed resistance to DOX, CARB, TMZ, and CIS. Additionally, the GBM cells demonstrated personalized sensitivity to the chemotherapy drugs. For instance, the GBM sample from patient 57595 was the most sensitive to DOX and TMZ. GBM samples from patients 15159, 7934, and 48993 and from 25873 and 7593 were most sensitive to CARB and CIS, respectively. The highest sensitivity to ETO was observed in GBM cells from patients 60886, 10677, and 10448. 

Notably, GBM cells from patients 7934, 1401, 10677, 10448, and 48993 and from 60886, 9439, 10448, 27980, and 12645 were also most sensitive to LL-37 and PG-1. Both LL-37 and PG-1 exhibited potent anticancer activities (IC_50_ values ranging from 1.0 to 35.6 μM), surpassing the efficacy of the chemotherapy drugs.

Subsequently, we assessed the effects of combinations of LL-37 and PG-1 with chemotherapy drugs on GBM cells by calculating the IC_50_ values for these combinations (Table 3 and Table 4).

Data presented in Table 3 and Table 4 reveal that GBM samples 11961, 6770, 25873, 55068, and 114495 showed increased sensitivity to the combination of LL-37 with DOX, compared to DOX alone. Similarly, GBM cells from patients 6770, 25873, 55068, and 60886 exhibited greater sensitivity to the combination of PG-1 with DOX. Combinations of LL-37 with carboplatin or TMZ were more effective than the individual chemotherapy drugs alone for GBM samples from 11081, 7934, and 60886. Additionally, GBM cells from patients 57595 and 55068 demonstrated enhanced sensitivity to the LL-37 and CARB combination, compared to CARB alone. GBM cells from patients 6770, 49142, and 114495 were more sensitive to the combination of LL-37 with TMZ than to TMZ alone. GBM samples from 11081, 25873, and 60886 also showed increased sensitivity to the combinations of PG-1 with DOX, TMZ, or CIS compared to the individual chemotherapy drugs. Notably, eight out of ten GBM samples were more responsive to the combination of PG-1 with TMZ than to TMZ alone. Furthermore, GBM samples from 11081, 6770, 57595, and 15159 exhibited greater sensitivity to the combinations of LL-37 with CIS or ETO, compared to the chemotherapy drugs alone. GBM cells from patients 25873 and 60886 responded more favorably to the LL-37 and CIS combination, while sample 7934 was more sensitive to LL-37 combined with ETO than to ETO alone.

Six out of ten GBM samples were more sensitive to the combination of PG-1 with CIS, compared to CIS alone. The combination of PG-1 with ETO was more sensitive for GBM cells from patients 6770, 7934, 25873, and 15159. In contrast, the GBM cells of the patients also showed resistance to the combined chemotherapy with LL-37 and PG-1, as shown in Table 3 and Table 4. However, resistance was also observed. GBM samples from patients 11081, 49142, 57595, and 62642 were more resistant to the combinations of LL-37 or PG-1 with doxorubicin. Additionally, patients 7934, 49142, 15159, and 60886 showed increased resistance to the LL-37 and doxorubicin combination, compared to doxorubicin alone. GBM cells from patients 11961, 25873, and 15159 exhibited greater resistance to the combinations of LL-37 with carboplatin or TMZ compared to the chemotherapy drugs alone. 

Moreover, GBM samples 6770 and 114495 exhibited greater resistance to the combination of LL-37 with carboplatin, compared to carboplatin alone. In contrast, GBM samples 7934, 49142, 57595, and 62642 demonstrated resistance to the PG-1 + CARB and PG-1 + CIS combinations. GBM cells from patients 57595 and 55068 were more resistant to the combination of LL-37 with TMZ than to TMZ alone. Interestingly, only two GBM samples, 15159 and 62642, showed resistance to the combination of PG-1 with TMZ compared to TMZ alone. Furthermore, GBM cells from patients 49142, 57595, and 62642 were more resistant to the combinations of LL-37 with CIS or ETO than to these drugs alone. The same patients, along with 57595, also exhibited increased resistance to the combinations of PG-1 with CIS or ETO, compared to the individual chemotherapy drugs. GBM samples 55068, 60886, and 7934 showed greater resistance to the combinations of PG-1 with ETO and CIS than to the drugs alone.

### 3.2. Prediction of GBM Patients’ Overall Survival Based on IC_50_ Values of Chemotherapy Drugs, LL-37, PG-1, and Their Combinations 

**Figure 1:**
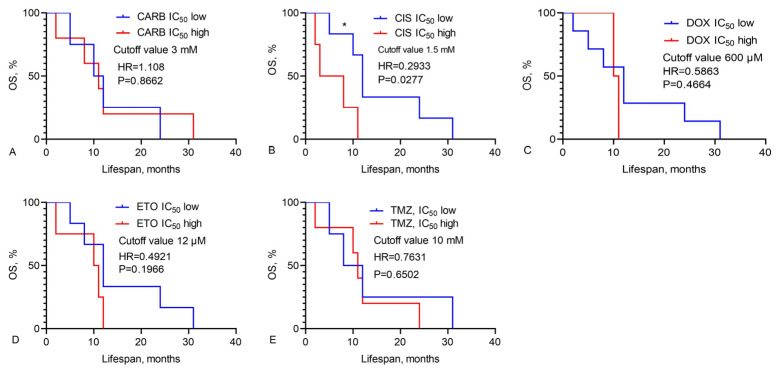
Overall survival of GBM patients, based on high and low IC_50_ levels of chemotherapy drugs. The OS of GBM patients was assessed according to their IC_50_ levels for various chemotherapy drugs: (**A**) carboplatin, (**B**) cisplatin, (**C**) doxorubicin, (**D**) etoposide, (**E**) temozolomide. The analysis was conducted using the Mantel–Cox test (χ^2^), with the following abbreviations: CARB (carboplatin), CIS (cisplatin), DOX (doxorubicin), ETO (etoposide), and TMZ (temozolomide). * Statistically significant (*p* < 0.05) differences high and low levels IC_50_ between groups.

**Figure 2:**
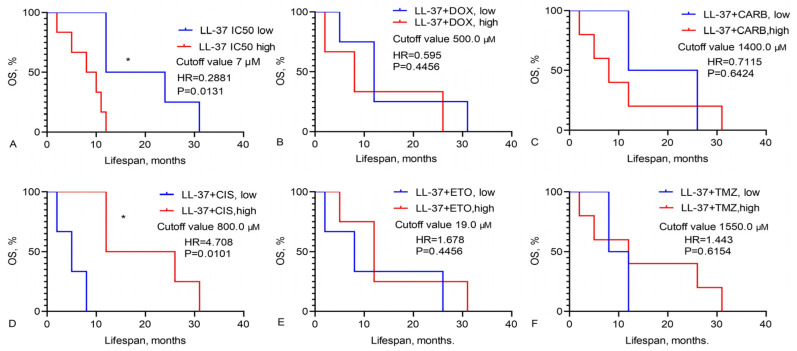
Overall survival of GBM patients, based on high and low IC_50_ levels of (**A**) LL-37 and its combinations with chemotherapy drugs: (**B**) doxorubicin, (**C**) carboplatin, (**D**) cisplatin, (**E**) etoposide, (**F**) temozolomide. Mantel–Cox test, χ^2^. * Statistically significant (*p* < 0.05) differences high and low levels IC_50_ between groups.

**Figure 3:**
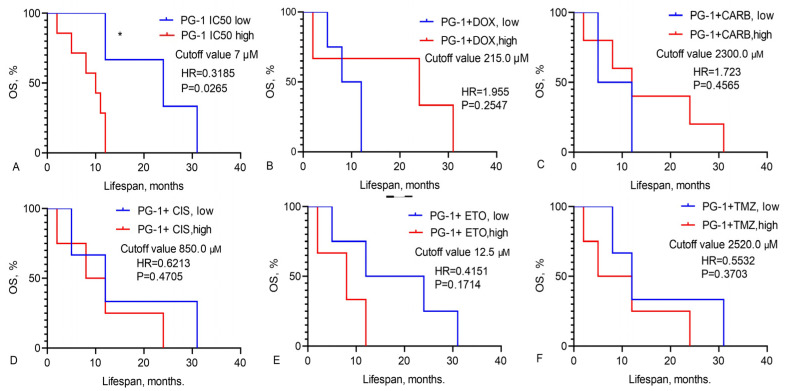
Overall survival of GBM patients based on high and low IC_50_ levels of (**A**) PG-1 and its combinations with chemotherapy drugs: (**B**) doxorubicin, (**C**) carboplatin, (**D**) cisplatin, (**E**) etoposide, (**F**) temozolomide. Mantel–Cox test, χ^2^. * Statistically significant (*p* < 0.05) differences high and low levels IC_50_ between groups.

We calculated the OS of GBM patients based on the IC_50_ values of chemotherapy drugs, LL-37, PG-1, and their combinations, as illustrated in Figure 1, Figure 2 and Figure 3.

Data in Figure 1 demonstrate that the median OS of GBM patients varies with different IC_50_ levels of chemotherapy drugs. For CIS, patients with a low IC_50_ (a cutoff value less than 1.5 mM) had a median OS of 12.0 months, whereas those with a high IC_50_ (a cutoff value greater than 1.5 mM) had a median OS of only 5.5 months (HR = 0.2933, χ^2^ = 4.849, *p* = 0.0277). 

Data presented in Figure 2 show that the median OS of GBM patients with a low IC_50_ of LL-37 (cutoff < 7 μM) was 18.0 months, compared to 9 months for those with a high IC_50_ of LL-37 (cutoff > 7 μM, HR = 0.2881, χ^2^ = 6.160, *p* = 0.0131) (Figure 2A). For GBM patients with a low IC_50_ for the LL-37 and CIS combination (cutoff < 800 μM), the median OS was only 5.0 months, whereas it was 19 months for those with a high IC_50_ value (cutoff > 800 μM, HR = 4.708, χ^2^ = 6.624, *p* = 0.0101) (Figure 2D).

Data in Figure 3 show that the median OS for GBM patients with a low IC_50_ of PG-1 (cutoff < 7 μM) was 24.0 months, whereas it was only 10 months for those with a high IC_50_ (cutoff > 7 μM, HR = 0.3185, χ^2^ = 4.923, *p* = 0.0265) (Figure 3A). 

### 3.3. Study of Survival Rates in Wistar Rats with a C6 Glioma Following Intranasal Administration of LL-37 and PG-1

**Figure 4:**
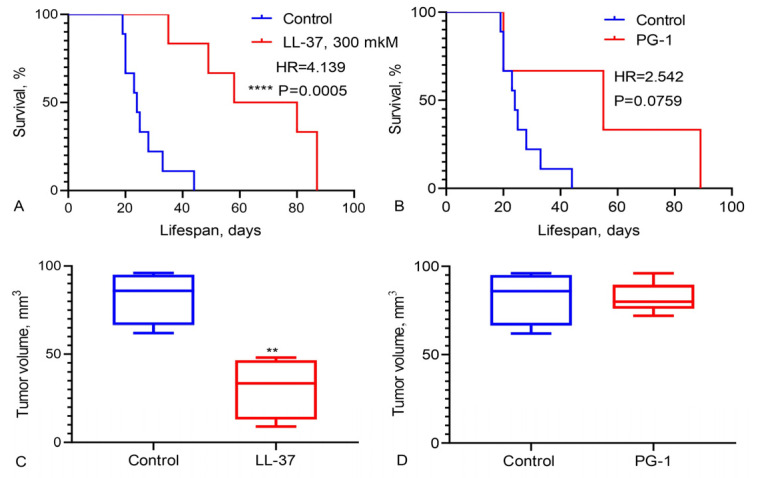
Overall survival of Wistar rats with C6 glioma following the administration of (**A**) LL-37 and (**B**) PG-1. Tumor volume following the administration of (**C**) LL-37 and (**D**) PG-1. The survival analysis was performed using the Mantel–Cox test with the χ^2^ values indicated. ** Statistically significant (*p* < 0.01) and **** (*p* < 0.0001) differences between tumor volume and overall survival rats under LL-37 administration from control group.

The observed high sensitivity of GBM cells to LL-37 and PG-1, as indicated by low IC_50_ levels, is statistically significantly associated with increased patient survival. Additionally, low IC_50_ values for the combination of PG-1 with ETO in GBM cells may correlate with enhanced patient longevity. Conversely, high IC_50_ levels for the combination of LL-37 with CIS in GBM cells also appear to be associated with extended survival in patients. These findings regarding the in vitro sensitivity of GBM cells to LL-37 and PG-1, and their correlation with patient survival, are supported by our experimental results on Wistar rats with C6 glioma. As illustrated in Figure 4, the survival rate of these rats was evaluated following the intranasal administration of LL-37 and PG-1.

Data in Figure 4 reveal that the median survival rate of rats with C6 glioma treated with LL-37 (at a dose of 300 μM) was 69 days, compared to 24 days in the control group (HR = 4.139, χ^2^ = 11.94, *p* = 0.0005) (Figure 4A). Similarly, the median survival rate of rats with C6 glioma treated with PG-1 (at a dose of 600 μM) was 55 days, while the control group median survival rate was 24 days (HR = 2.542, χ^2^ = 3.151, *p* = 0.0759), Figure 4B. Additionally, LL-37 treatment significantly reduced the volume of C6 glioma in the rats.

## 4. Discussion

In this study, we demonstrated that the cationic antimicrobial peptides LL-37 and PG-1 exhibit potent anticancer effects against GBM cells (Table 3 and Table 4). Previous research has shown that LL-37 secretion levels and *CAMP* gene expression are significantly reduced in colorectal cancer, glioma, and SH-SY5Y neuroblastoma [25,28,37,38]. Low expression of the *CAMP* gene could serve as an important biomarker for these cancers. LL-37’s mechanisms of action involve interactions with various receptors, including formyl-peptide receptor-2 (FPR2), C-X-C motif chemokine receptor 2 (CXCR2), purinergic receptors P2Y11 and P2X7, MAS-related GPR family member X2 (MrgX2), EGFR/ErbB1, erb-b2 receptor tyrosine kinase 2 (ERBB2), ligand-gated ion channels (LGIC), insulin-like growth factor 1 receptor (IGF1R), and toll-like receptors (TLRs), which are often overexpressed in tumors compared to normal cells. LL-37 typically binds to the transmembrane regions of these receptors, leading to conformational changes and the modulation of receptor activity [19,39,40,41,42,43]. The binding of LL-37 to one of the G-protein-coupled receptors, although not yet fully characterized, results in decreased Bcl-2 levels and induces a cascade of apoptotic proteins, including Bax, Bak, Puma, and p53, as well as endonuclease G (EndoG) in HCT116 colorectal cancer cells [38].

Additionally, the expression of the *CAMP* gene in various cancer cells is inducible, for instance, by vitamin D3 [27]. This underscores the importance of further research to explore the relationship between *CAMP* expression and tumor development. The potential for LL-37 and its derivatives as therapeutic agents against malignant neoplasms has been extensively discussed in the literature. Several analogs of LL-37 with demonstrated antitumor activity have been developed and patented, highlighting the peptide’s promising role in cancer treatment.

The mechanisms underlying PG-1’s actions against tumor cells remain underexplored. Experimental data indicate that PG-1 disrupts the cellular membranes. It has been shown to exhibit cytotoxic effects against various human cancer cell lines, including K562 erythromyeloid leukemia, U-937 lymphoma, A-549 and A-431 epithelioid lung carcinomas, MG-63 osteosarcoma, and SH-SY5Y neuroblastoma cells [44,45,46]. Specifically, in MCF-7 cells, PG-1’s cytotoxicity is linked to its oligomerization within cell membranes. This oligomerization leads to the formation of transmembrane pores, resulting in intracellular Ca^2+^ influx and the inhibition of cyclin-dependent kinase 1A (CDKN1A) and proliferating cell nuclear antigen (PCNA). This cascade of events ultimately triggers apoptosis via p53 protein and caspase-3 activation [47,48]. CDKN1A and PCNA inhibit the transition from the G1 to the S phase of the cell cycle, thus blocking cancer cell division and tumor growth [49]. PG-1 interacts with the transmembrane domains of MrgX2, IGF1, and EGF receptors [50,51], leading to the upregulation of p53 and apoptotic genes such as CDKN1A and p21 [49]. 

The cytotoxic effect of recombinant PG-1 on SH-SY5Y neuroblastoma cells is correlated with the presence of anionic sulfated proteoglycans in the tumor cell membranes. Notably, the membranes of malignant SH-SY5Y neuroblastoma cells exhibit a higher negative charge compared to low-grade tumor cells, which may facilitate the transport of K^+^ and Ca^2+^ ions into the cells. This alteration in transmembrane potential enhances PG-1 binding to these membranes [46]. These findings partially elucidate PG-1’s selective anticancer activity against neuroblastoma cells, as opposed to non-neuronal cells like NIH-3T3 and HEK293T fibroblasts. Recent MTT assays and trypan blue staining have demonstrated that both PG-1 and LL-37 exhibit potent anticancer effects at concentrations below 10^−5^ M, showing greater efficacy compared to conventional chemotherapy drugs in C6 and U251 glioma cells [29,30].

We assessed the effects of combining LL-37 and PG-1 with various chemotherapy drugs on GBM cells from patients (Table 3 and Table 4). The combinations of LL-37 and PG-1 with CIS, ETO, and PG-1 with TMZ showed lower IC_50_ values compared to the IC_50_ levels of the chemotherapy drugs when used alone. These findings align with the observation that these specific combinations of LL-37 with cisplatin and PG-1 with etoposide are associated with improved survival and OS in GBM patients, as shown in Figure 2 and Figure 3. Furthermore, these combinations also resulted in an increased lifespan for rats with C6 glioma following the intranasal administration of LL-37 and PG-1, as illustrated in Figure 4. 

Our recent studies have demonstrated that combinations of PG-1 and LL-37 with DOX and CARB exhibit synergistic effects in GBM cells [37]. Using the MTT assay, we identified synergistic effects for the combinations of PG-1 with CARB and LL-37 with ETO, and an additive effect for the combination of PG-1 with TMZ in C6 glioma cells. Additionally, trypan blue staining revealed that combinations of PG-1 with DOX and CIS showed synergistic cytotoxic effects on C6 glioma cells [29]. LL-37 also synergistically enhanced the effects of DOX and gentamicin in human K562 erythromyeloid leukemia cells by increasing the ratio of necrotic and apoptotic cells. For PG-1, synergy was observed in combination with doxorubicin, actinomycin D, or polymyxin B in K562 cells, inducing both necrosis and apoptosis [44]. 

Both PG-1 and LL-37 enhance the anticancer effects of chemotherapy by damaging cell membranes and/or penetrating intracellularly, demonstrating a more pronounced effect on tumor cells compared to healthy cells. Consequently, PG-1 may be considered a more promising candidate for combination therapy, compared to LL-37. To further improve the selectivity of PG-1 toward tumor cells, various strategies could be employed, such as designing new structural analogs of the peptide with optimized properties or creating chimeric molecules that incorporate the protegrin sequence, along with specific binding sites for tumor cell markers. 

In contrast, it is known that anticancer immunity is provided by cytotoxic T- and B-lymphocytes, representing plasma cells and their antibodies [52,53,54]. T- and B-lymphocytes, like other blood cells, form the immune microenvironment of the GBM [55]. Human cathelicidin LL-37 is a chemoattractant molecule for neutrophils, monocytes, macrophages, mast cells, and T-lymphocytes, and promotes the mobilization of immune cells in the adaptive immune response phase. All these immune cells express formyl peptide receptor-1 (FPRL-1), to which LL-37 selectively binds [56]. It is possible that LL-37 activates T- and B-lymphocytes and the secretion of antibodies, which will enhance their antiviral and anticancer immunity, ensuring an increase in the life expectancy and survival of patients and rats [57]. However, the detailed molecular mechanisms by which PG-1 and LL-37 exert their effects on GBM cells remain inadequately explored, highlighting the need for continued research in this area.

The main limitation of this study is the small size of the GBM patients sample (*n* = 27). This investigation did not include all brain tumors, recurrent, and non-operated GBMs, but used only the primary GBM samples. GBM samples were obtained from one medical center (Almazov National Medical Research Centre) in Saint Petersburg in 2021–2024.

## 5. Conclusions

LL-37 and PG-1 exhibit strong in vitro anticancer effects that surpass those of traditional chemotherapy drugs against GBM cells. The anticancer efficacy of LL-37 and PG-1 is significantly correlated with increased life expectancy and OS in GBM patients. This correlation is further supported by experiments showing that the intranasal administration of LL-37 and PG-1 significantly extends the lifespan of rats with C6 glioma. Our study also evaluated the effects of combining LL-37 and PG-1 with chemotherapy drugs on GBM cells from patients. We found that both low and high IC_50_ values for the combinations of PG-1 with etoposide and LL-37 with cisplatin were associated with increased life expectancy and OS in GBM patients. These promising results suggest that such combinations could be developed for future GBM treatments. Overall, these findings underscore the importance of further research into the mechanisms of LL-37, PG-1, and their combinations as potential therapies for brain tumors.

## Figures and Tables

**Table 1:** Doses of the LL-37, PG-1, and chemotherapy drugs.

Drugs	Dose, μM
Doxorubicin	920.0, 460.0, 230.0, 115.0, 73.6, 36.8, 18.4
Carboplatin	26,900.0, 2690.0, 1350.0, 673.0, 269.0, 134.0
Cisplatin	1660.0, 830.0, 332.0, 166.0, 83.0, 33.2, 16.1
Temozolomide	15,500.0, 5150.0, 1550.0, 773.0, 386.0, 155.0
Etoposide	27.0, 13.5, 6.7, 3.3, 1.6, 0.8
LL-37	32.0, 16.0, 8.0, 4.0, 2.0, 1.0
PG-1	64.0, 32.0, 16.0, 8.0, 4.0, 2.0

Note: LL-37, PG-1, and chemotherapy drugs were dissolved in DMEM at IC_50_.

**Table 2:** The IC_50_ of the chemotherapy drugs, LL-37, and PG-1 in human glioblastoma cells, according to an MTT assay.

ID Patient	IC50, μM						
	DOX	CARB	TMZ	CIS	ETO	LL-37	PG-1
11081	290.4	29,431.0	16,179.5	2448.4	27.0	10.3	16.0
11961	3350.3	39,792.9	43,539.3	11,919.7	86.5	32.2	123.6
6770	850.0	4000.0	14,000.0	1090.0	26.3	9.5	8.7
7934	50.9	2000.0	7491.0	200.0	7.5	2.0	1.2
49142	548.3	2708.4	11,056.0	776.0	11.4	6.6	7.4
25873	560.0	888.8	8619.2	300.0	8.9	24.1	30.1
57595	16.9	3093.6	194.5	1682.3	7.5	8.3	8.6
55068	546.5	27,574.5	4789.5	1104.8	11.8	6.4	3.9
15159	179.2	116.4	436.8	698.1	11.4	32.1	15.8
62642	20.3	42,495.1	24,015.7	1158.5	32.3	28.1	34.3
60886	278.8	4498.0	2174.3	>1660.0	6.3	1.1	1.2
18871	2682.8	24,031.9	11,976.9	1776.4	30.9	24.3	23.8
114495	3350.3	39,792.9	43,539.3	965.8	86.5	26.8	35.4
10677	1180.1	20,471.8	1309.1	2448.4	3.4	3.5	7.4
1401	920.0	5136.5	611.8	261.2	10.3	4.0	16.0
18871	2682.8	24,031.9	11,976.9	1776.3	30.9	24.3	23.8
8989	817.1	20,195.2	14,486.0	1218.8	26.3	9.7	12.1
20939	920.0	17,861.9	15,500.0	476.5	38.0	11.8	19.3
39114	3458.6	25,000.0	12,282.1	1824.2	32.8	26.8	26.2
40906	1083.2	38,147.6	14,961.7	1596.1	58.9	20.8	19.4
48993	–	1126.8	15,407.5	120.4	38.7	3.1	14.1
48307	1260.3	20,852.7	1510.7	1280.8	9.5	5.7	6.2
9439	1513.2	26,116.5	22,206.3	1784.9	41.3	7.2	0.8
10448	478.7	24,237.2	14,659.1	1299.0	3.4	1.9	4.3
27980	733.4	2223.4	5345.6	835.3	9.3	17.5	1.9
12645	483.6	2605.4	5258.3	729.8	7.0	7.2	3.9
7593	1123.9	2110.4	14,905.5	298.9	26.8	8.6	13.2

Note: DOX, doxorubicin; CARB, carboplatin; TMZ, temozolomide; CIS, cisplatin; ETO, etoposide; LL-37, human cathelicidin; PG-1, pig protegrin-1.

**Table 3:** The IC_50_ of the combinations of LL-37 with chemotherapy drugs in human GBM, according to an MTT assay.

ID Patient	IC_50_ with Combinations of LL-37 with Chemotherapy, in μM				
	DOX	CARB	TMZ	CIS	ETO
11081	1832.7	1645.3	5150.0	485.3	13.8
11961	227.2	55,122.9	54,577.5	4825.5	12.7
6770	160.1	26,481.5	1507.7	790.5	14.2
7934	160.0	1360.0	1550.0	800.0	19.5
49142	989.4	27,187.6	10,247.5	1594.8	11.8
25873	15.1	3307.9	9580.8	238.3	40.0
57595	4852.6	1424.0	689.6	388.2	6.6
55068	262.5	1234.5	5993.7	1201.9	37.4
15159	563.5	24,100.4	5494.4	634.2	2.89
62642	91.9	48,281.2	24,523.8	984.5	37.7
60886	910.7	742.5	365.1	919.3	12.6
114495	178.9	35,771.3	37,621.4	351.9	0.96

Note: Green indicates that the IC_50_ values of the combinations of LL-37 with chemotherapy drugs are lower than the IC_50_ values of the chemotherapy drugs presented in Table 2. Red indicates that the IC_50_ values of the combinations are higher than the IC_50_ values of chemotherapy drugs shown in Table 2. IC_50_ values for combinations that are not specifically highlighted in Table 3 or Table 4 are comparable to the IC_50_ values of chemotherapy drugs presented in Table 2.

**Table 4:** The IC_50_ values of the combinations of PG-1 with chemotherapy drugs in human GBM, according to an MTT assay.

ID Patient	IC_50_ the Combinations of PG-1 with Chemotherapy, μM				
	DOX	CARB	TMZ	CIS	ETO
11081	1380.4	23,637.9	15,166.3	1118.6	37.1
6770	219.4	23,963.5	4424.8	750.4	6.4
7934	50.0	2269.4	2518.5	700.0	0.9
49142	1230.3	32,964.1	6117.6	1575.4	12.5
25873	74.9	362.2	4980.5	59.4	0.8
57595	41.2	14,674.4	2.8	3851.7	16.6
55068	215.8	37,804.8	1698.3	840.6	14.2
15159	179.1	1648.5	5297.4	315.3	4.7
62642	2299.2	44,725.1	38,364.4	3649.8	73.5
60886	30.6	3312.5	375.5	2295.3	25.8

Note: Green indicates that the IC_50_ values of the combinations of PG-1 with chemotherapy drugs are lower than the IC_50_ values of chemotherapy drugs presented in Table 2. Red indicates that the IC_50_ values of the combinations are higher than the IC_50_ values of chemotherapy drugs shown in Table 2. IC_50_ values for combinations that are not specifically highlighted in Table 3 or Table 4 are comparable to the IC_50_ values of chemotherapy drugs presented in Table 2.

## Data Availability

The data presented in this study are openly available on Figshare at 10.6084/m9.figshare.16879432.
